# Suspected Focal Junctional Tachycardia with Type I Isorhythmic Atrioventricular Dissociation Associated with Diffuse Lymphocytic Myocarditis of Undetermined Etiology in a Cat

**DOI:** 10.3390/ani16213437

**Published:** 2026-10-30

**Authors:** Iván Miralles-Plaza, Marina Martín-Ambrosio-Francés, Isabel del Portillo-Miguel, Hayley-Rebecca McDonald, Altin Cala, Inmaculada Cerrada-Serra

**Affiliations:** 1 Veterinary Teaching Hospital, Universidad Cardenal Herrera CEU, CEU Universities, Calle Santiago Ramón y Cajal s/n, 46115 Valencia, Spain; inmaculada.cerrada@uchceu.es; 2 University Veterinary Teaching Hospital, Faculty of Biomedical and Health Sciences, Campus de Villaviciosa, Universidad Europea de Madrid, Calle Tajo s/n, 28670 Madrid, Spain; marina.martin-ambrosio@universidadeuropea.es; 3 Small Animal Hospital Royal (Dick) School of Veterinary Studies, the University of Edinburgh, Roslin EH25 9RG, UK; idelportmig@gmail.com; 4 Independent Researcher, San Francisco, CA 94110, USA; hayley.mcdonald1@uq.net.au; 5 Independent Researcher, Marlow SK7 1DX, UK; altincala73@gmail.com; 6 Department of Animal Medicine and Surgery, Universidad CEU-Cardenal Herrera, CEU Universities, Calle Assegadors, 2, 46115 Valencia, Spain

**Keywords:** focal junctional tachycardia, isorhythmic atrioventricular dissociation, heart failure, myocarditis, cats

## Abstract

Focal junctional tachycardia (FJT) is a regular, narrow QRS complex tachycardia with subtle cycle length irregularity, frequently associated with isorhythmic atrioventricular dissociation (IAVD). Its presentation may be paroxysmal or non-paroxysmal. This rhythm is well recognized in humans and dogs. However, to the best of the authors’ knowledge, FJT has not been previously published in cats. This case report describes a 9-year-old neutered male domestic shorthair cat presenting with signs of congestive heart failure, hyperthermia, and a non-paroxysmal FJT. Electrocardiography demonstrated a narrow QRS tachycardia with type I synchronization IAVD, consistent with suspected FJT. Transthoracic echocardiography revealed severe biatrial enlargement, concentric ventricular hypertrophy and echocardiographic measures suggestive of high probability of pulmonary hypertension. There was apparently preserved global systolic function and diastolic dysfunction. These findings were consistent with a non-specific cardiomyopathy phenotype stage C. Pleural fluid cytology initially suggested a lymphoproliferative disorder; however, postmortem histopathological examination confirmed diffuse lymphocytic myocarditis without evidence of neoplasia. Despite intensive supportive and antithrombotic therapy, the cat experienced sudden death. To the authors’ knowledge, this is the first description of suspected FJT with type I IAVD in a cat. This case highlights myocarditis as a critical differential diagnosis in cats presenting with a lymphocytic effusion, structural echocardiographic changes, and complex supraventricular arrhythmias. It also discusses the diagnostic challenges and prognostic implications associated with inflammatory myocardial disease in this species.

## Simple Summary

Focal junctional tachycardia with type I isorhythmic AV dissociation is a rapid, abnormal heart rhythm—where the upper and lower chambers of the heart beat independently of one another. This rhythm is well recognized in humans and dogs, but has never been reported in cats. This report aims to describe the first known case of this dangerous rhythm in a nine-year-old cat experiencing heart failure and fever. Diagnostic tests revealed an arrhythmia, enlarged cardiac chambers, and thickened heart muscle. Furthermore, analysis of the pleural effusion was suspicious for neoplasia. Despite intensive medical care, the cat passed away suddenly. A tissue examination after death revealed severe inflammation of the heart muscle. We conclude that severe heart inflammation can cause this rare heart rhythm and mimic cancer in feline patients. This finding expands our understanding of this condition in cats, and alerts veterinarians to consider heart inflammation as an uncommon, but possible, differential diagnosis. This manuscript also discusses management of a complex, life-threatening arrhythmia.

## 1. Introduction

Focal junctional tachycardia (FJT) is a narrow QRS complex tachycardia resulting from enhanced automatism or triggered activity in the junctional area, proximal to the apex of Koch’s triangle and posterior to the distal atrioventricular (AV) bundle and the compact AV node [1]. FJT has been reported in young adult Labrador Retrievers who may or not manifest clinical signs of such episodic weakness [2]. In human patients, it may be congenital [3,4], postoperative [5], or linked to systemic conditions (i.e., enterovirus myocarditis, Lyme’s disease, digitalis toxicity) [6,7,8]. In both humans and dogs, FJT is typically drug-resistant and can lead to tachycardia-induced cardiomyopathy (TICM), right and/or left congestive heart failure (CHF) and even cardiac arrest [2,7,8]. In dogs, this arrhythmia exhibits a median heart rate (HR) of 140 beats/min with median QRS duration of 52 ms and, usually, a regular ventricular cycle length of 420 ms [2].

FJT often displays isorhythmic atrioventricular dissociation (IAVD) [4], meaning that the atria and ventricles are driven by independent pacemakers at equal or nearly equal rates [2]. Experimental studies demonstrated that when separate and distinct cellular elements having no anatomic continuity and possessing inherently different rhythms are placed in contact with each other, they begin to discharge impulses simultaneously at a common rate [9]. Furthermore, when two independent foci of impulse formation exist side by side, they can interact with each other in such a way that the focus with the lower rate of discharge accelerates to approximate that with the higher frequency [9]. These phenomena are termed ‘synchronization’ when the two independent rhythms occur for a long period, or ‘accrochage’ when lasting for only a few beats [9].

IAVD is described as two patterns of synchronization: type I is characterized by a rhythmic fluctuation of the P-QRS complexes, most often with the P waves oscillating gradually back and forth across the QRS complexes with periodically varying PQ intervals. At times, the P waves may be embedded in the QRS complexes or appear immediately after; however, because the difference between the two pacemaker discharge rates is very small, they are not usually seen after the descending branch of the R waves in the ST segment, or on top of the T waves [2]. Conversely, in type II synchronization (or ‘capture synchronization’), the sinus P waves and junctional QRS complexes remain in a fixed position with respect to each other [1]. If the automatic discharge rate of the AV junction is faster than that of the sinus node, the junctional wavefront is conducted retrograde to the atria via the AV nodal fast pathway and 1:1 ventriculoatrial conduction ensues [2]. In such cases, sinus P waves are absent and retrograde concentric atrial activation occurs. This is seen by P’ waves that are negative in the inferior leads (II, III and aVF), isodiphasic in lead I, and equally positive in aVR and aVL with a mean electrical axis (MEA) between −80° and +100° [2]. These P’ waves are usually inscribed in the descending branch of the R waves or in the first portion of the ST segment, with a short RP’ interval mimicking an S wave (pseudo-S wave) [2]. Non-paroxysmal FJT shows gradual onset and termination characterized by the warm-up and cool-down phenomena, short P’Q interval, and a 1:1 AV conduction ratio [1]. IAVD with accrochage has only been described in a dog with ruptured chordae tendinae [10], whilst synchronization has been described in one canine patient with suspected AV junctional tachycardia [11]. A more current case series in 11 Labrador Retrievers undergoing electrophysiologic studies, revealed a correlation between IAVD and FJT [2]. In cats, atrioventricular (AV) dissociation has been described in accelerated idioventricular rhythm and ventricular tachycardia [12], ventricular pacing [13], ventricular pre-excitation (VPE) [14,15,16], and paroxysmal high-grade second-degree and persistent third-degree AV blocks [17] either associated with systemic, underlying cardiac disease [18], or even in the absence of structural heart disease [14]. Additionally, transient IAVD has been reported in cats linked to hypothermia [19,20], normothermia [21] and anesthesia [22].

Primary hypertrophic cardiomyopathy (HCM) followed by restrictive (RCM) and non-specific (NSCM) cardiomyopathies are the most common heart diseases in cats leading to ventricular or supraventricular arrhythmias, CHF and thromboembolism [23]. Secondary cardiomyopathies such as hypertensive cardiopathy [24], transient myocardial thickening, myocarditis [25,26] or, rarely, some infiltrative diseases such as cardiac lymphoma affecting either the myocardium [27] and/or pericardium [28] can mimic HCM.

Inflammatory cardiomyopathies in cats have been poorly described. Myocarditis is defined by the presence of a focal or diffuse inflammatory cell infiltration of the myocardium which has a high risk of deteriorating cardiac function [29]. Causes of myocarditis in cats have included viral pathogens such as feline coronavirus (FCoV), as part of the feline infectious peritonitis (FIP) syndrome and feline immunodeficiency virus (FIV) [30,31,32,33]. Other infections have also been described, including *Bartonella henselae* [34,35], *Streptococcus canis* [36], *Toxoplasma gondii* [37,38], *Sarcocystis felis* [39] and *Trypanosoma cruzi* [40]. In dogs, a broad spectrum of infectious etiologies is recognized, similar to humans [29]. These include bacterial, viral or protozoal agents [41,42,43]. In humans, although the most frequent causes are viral (coronavirus SARS-CoV2, HIV) and rheumatic, an immune-mediated form of cardiotoxicity-induced myocarditis in patients receiving targeted cancer therapies has been well documented [44]. However, diagnosing myocarditis remains challenging, with the cause unknown in up to 80% of cases in human patients [45].

Clinically, myocarditis in humans [45], dogs and cats, can present as an acute (<2 weeks), subacute (2–4 weeks) or chronic (>4 weeks) process exhibiting malignant arrhythmias, CHF and even cardiac arrest [33,41,45]. In cats, it is commonly manifested with overt CHF signs such as respiratory distress due to serohemorrhagic pleural or pericardial effusions often refractory to standard therapy, ventricular arrhythmias, aortic thromboembolism (ATE) and pyrexia above 39.6 °C [41,42,43]. Since myocarditis is a histopathological finding, the antemortem diagnosis must be confirmed by endomyocardial biopsy when feasible [46]. In veterinary medicine, myocarditis is presumptively diagnosed based on clinical presentation supported by ancillary tests, including PCR testing, serology [34,46], circulating cardiac troponin I (cTnI) concentrations and echocardiographic findings consistent with myocardial dysfunction [47]. Although some major and minor criteria for the antemortem clinical diagnosis have been proposed in dogs, confirmation is often achieved postmortem due to its poor prognosis [41].

In cats, the clinical presentation, echocardiographic findings, and electrocardiographic features of inflammatory myocardial diseases, such as myocarditis and myocardial lymphoma, have been described primarily in small case series and individual case reports. However, to the best of the authors’ knowledge, complex arrhythmias associated with these conditions have rarely been reported in cats.

This publication aims to provide a detailed description of suspected FJT in a cat with echocardiographic evidence of NSCM phenotype, exhibiting dyspnea due to refractory lymphocytic pleural and pericardial effusions associated with underlying myocarditis.

## 2. Case Presentation

A 9-year-old, male neutered, domestic shorthair cat weighing 8 kg was admitted for investigation of progressive respiratory distress of a duration of two weeks. The patient was strictly indoors and preventive medications were up to date. The cat had been overweight for several years and was fed a high-quality diet.

On admission, the patient appeared lethargic and obtunded, with a body condition score (BCS) of 4/5. His oral mucous membranes were cyanotic with a capillary refill time of 2 s. He was tachypneic at rest (80 breaths per minute) with open mouth breathing upon gentle manipulation. His cardiac auscultation revealed a HR of 174 beats/min, muffled heart sounds and continuous gallop sound with an irregular rhythm based on the presence of abrupt pauses and asynchronous femoral pulses on palpation. A positive hepatojugular reflux was elicited on abdominal palpation. The rectal temperature was 40 °C, consistent with hyperthermia. 

Given the presence of respiratory distress, cyanosis, a positive hepatojugular reflux, pulse deficits, and abnormal cardiac auscultatory findings, the presenting signs were suspected to reflect reduced cardiac output secondary to underlying cardiac disease, and an increase in right atrial pressure. The most likely differential diagnoses were cardiogenic shock secondary to structural heart disease, such as RCM, NSCM, arrhythmogenic right ventricular cardiomyopathy (ARVC) or hypertrophic cardiomyopathy (HCM) with or without concurrent pulmonary hypertension (PH). Pericardial diseases such as constrictive pericarditis or pericardial effusion with cardiac tamponade were also considered. Given the hyperthermia, inflammatory or infectious causes such as viral (FIV, feline leukemia virus [FeLV], parvovirus), bacterial (*Bartonella henselae*, *Streptococcus canis*) or protozoal myocarditis (*Toxoplasma gondii*, *Sarcocystis felis*, *Leishmania infantum*), pericarditis, or physiologic stress were included. Other systemic diseases such as neoplasia, either metastatic or primary (e.g., lung tumor, myocardial lymphoma), or endocrinopathies (e.g., hyperthyroidism, acromegaly) were considered less likely given the acute presentation, absent dermatological signs, normal micturition pattern and BCS. Toxic or coagulopathic disorders were also deemed unlikely based on his medical history.

Flow-by oxygen was supplemented on presentation due to suspected systemic hypoxemia. Mild sedation with butorphanol at a dose of 0.25 mg/kg was administered IM. Peripheral venous catheterization was performed as soon as the patient allowed manipulation. Point-of-care ultrasound (POCUS) confirmed the presence of both severe bilateral pleural and moderate circumferential pericardial effusions with early signs of cardiac tamponade. Non-invasive systolic blood pressure (NIBP) measurements by Doppler, were consistent with borderline hypotensive (100 mmHg, Ref. 110–190) [48].

**Figure 1:**
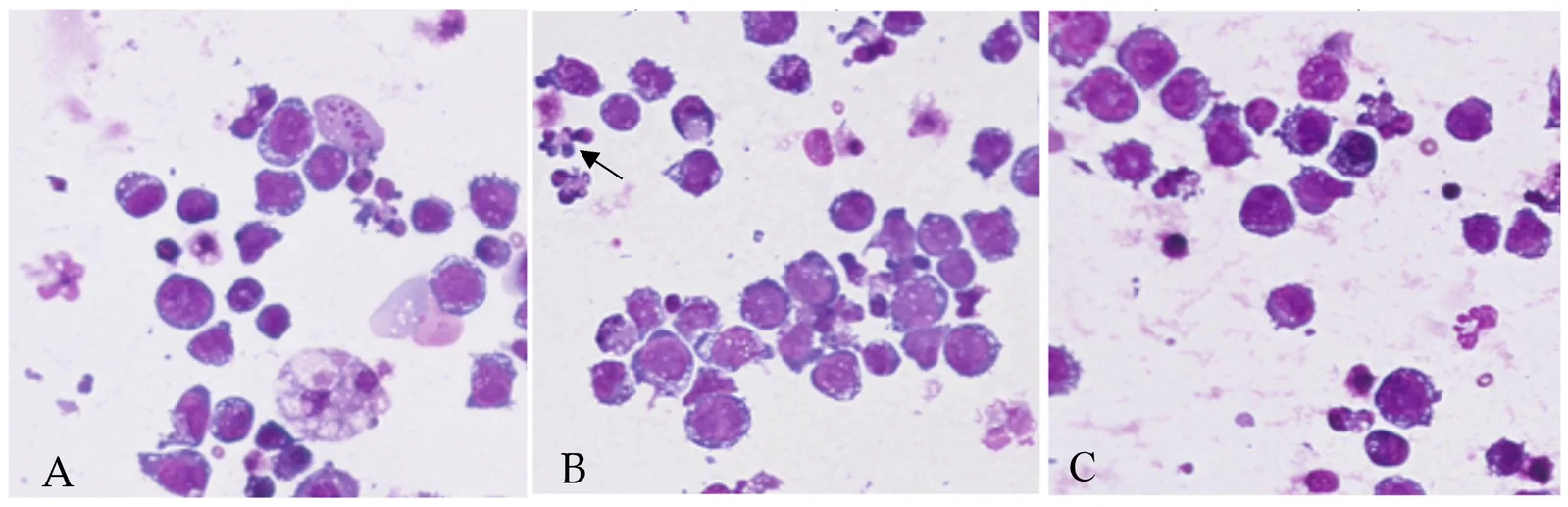
Microscopic evaluation of pleural fluid (**A**–**C**). High-power photomicrographs demonstrating a highly cellular preparation with well-preserved cellularity against a pale background. A homogeneous lymphoid population (>90% of cells) dominates the fields, consisting predominantly of intermediate-to-large-sized lymphocytes mixed with low numbers of macrophages and neutrophils. The intermediate-to-large-sized lymphocytes display rounded nuclei with finely stippled chromatin and frequently contain a prominent nucleolus. The cytoplasm is scant to moderate in amount and deeply basophilic, frequently containing small, clear vacuoles. Mitotic figures are observed across panels (arrow). (Diff-Quik stain, 1000× magnification/100× oil immersion objective).

Ultrasound-guided bilateral thoracocentesis was performed with 220 mL of viscid, amber-colored fluid drained from the pleural cavity. Fluid analysis revealed a protein-rich (3.0 g/dL of total solids, TS), dense and highly cellular (14,980.0 cells/μL) sample, consistent with an exudate. Fluid cytology was consistent with an exudative sterile effusion that was presumably neoplastic, highly suggestive of an intermediate-to-large-sized cell lymphoma (Figure 1). Immunophenotyping for lymphoma-type characterization was requested.

After initial stabilization, the resting respiratory rate (RRR) and effort significantly improved. Mucous membranes and systolic NIBP returned to normal (132 mmHg). Thoracic radiographs were performed post-thoracocentesis to assess for interstitial lung, bronchial, pleural or mediastinal disease. Radiographs revealed a scant amount of focal pleural effusion, cardiomegaly (VHS 9.4, Ref. 7.5 ± 0.3) [49] with prominent pulmonary veins suggestive of venous congestion without obvious lung infiltrates. 

**Figure 2:**
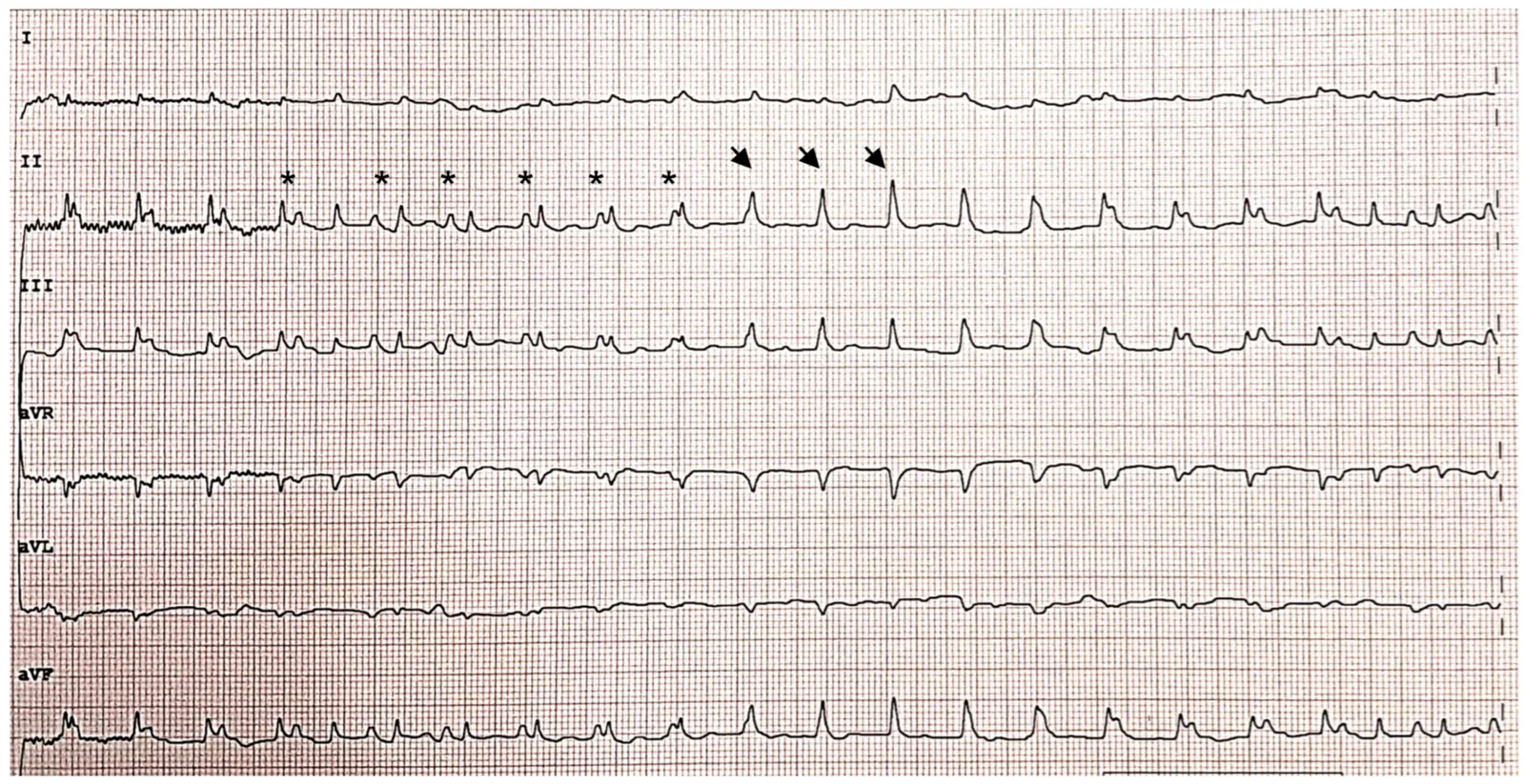
Surface 6-lead ECG at presentation. The trace showed a continuous fluctuation of the distance between atrial (AD) and ventricular depolarisations (R wave) resulting in a periodic variation of AD-R intervals and R-AD intervals (* on lead II). The P waves move closer to the QRS complexes, leading to shortening of the AD-R intervals until both overlap (arrows). Baseline artifacts can be observed during the first three cardiac beats. Paper speed: 50 mm/s, voltage: 20.0 mm/mV.

A six-lead surface ECG was recorded in order to characterize the arrhythmia with the patient positioned in right lateral recumbency. This showed a narrow QRS complex tachycardia (20 ms, upper reference limit (URL) 40 ms) with a mean HR of 260 beats/min. The mean electrical axis was normal (70°, reference from 0° to 160°). The P waves were of normal amplitude (0.2 URL < 0.2 mV) and duration (20 ms, URL < 40 ms). The cycle length (R-R intervals) displayed minimal cycle length irregularity (230 to 260 ms). Premature beats were not observed for arrhythmia initiation. Electrocardiographic findings were consistent with FJT with IAVD and type I synchronization during periods of suspected variability in atrial-Hisian (AH) intervals (Figure 2).

**Figure 3:**
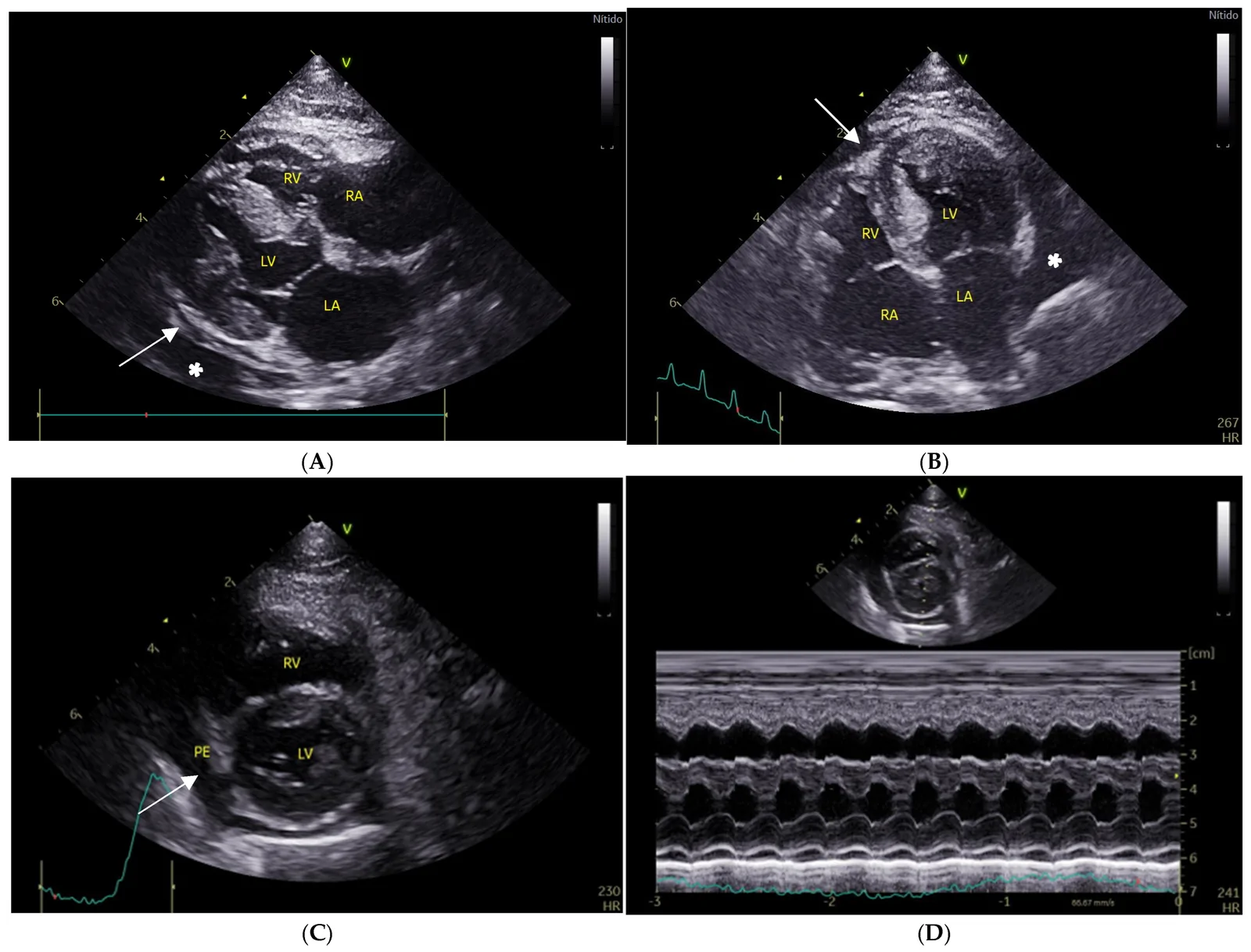
Transthoracic echocardiography after initial stabilization. The top images were obtained on two-dimensional mode from right parasternal longitudinal 4-chamber view (**A**) and left apical 4-chamber view (**B**), revealing left ventricular hypertrophy and a left ventricular band between the septal papillary muscle bridging to the left ventricular free wall. Both pleural (*) and pericardial effusions (arrows) are noted. The bottom images were obtained from right parasternal short-axis view at the level of the papillary muscles from bidimensional mode (**C**) and M-mode (**D**), showing mixed RV eccentric and concentric hypertrophy and dilatation, as well as the presence of pericardial effusion.. LA, left atrium; LV, left ventricle; RA, right atrium; RV, right ventricle. LV, left ventricle; RV, right ventricle; PE, pericardial effusion.

Transthoracic echocardiography (Figure 3) was performed after stabilization of the patient. The exam revealed severe biatrial enlargement with marked left atrial (LA) dilatation and spontaneous echocontrast within the LA appendage. Moderate right ventricular (RV) dilatation with concomitant RV free wall thickening was observed. This was considered likely secondary to pressure overload due to pulmonary hypertension (PH), although myocarditis and other infiltrative or neoplastic myocardial diseases were also considered (Figure 3). The left ventricle (LV) showed mild-to-moderate symmetric concentric hypertrophy. One left ventricular band was identified. The papillary muscles appeared prominent and diffusely hyperechoic. Minimal residual pleural and pericardial effusion was present, without evidence of cardiac tamponade. No mediastinal mass was detected.

**Figure 4:**
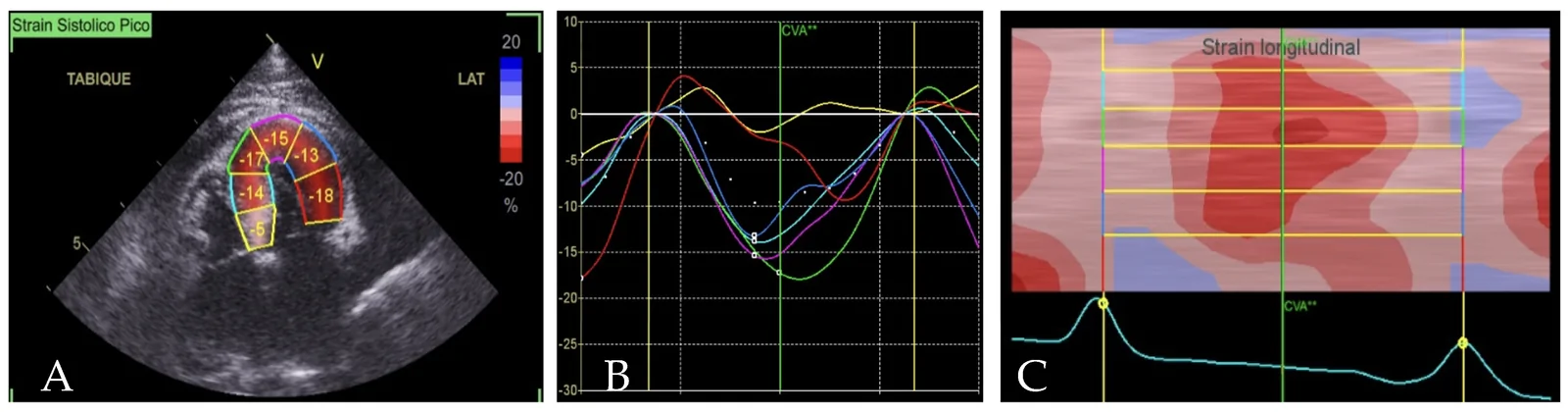
Two-dimensional speckle-tracking echocardiographic assessment of left ventricular longitudinal strain in this patient. (**A**) Peak systolic longitudinal strain values for individual left ventricular segments. A focal segment shows severely reduced systolic strain (−5%), while adjacent segments demonstrate moderately reduced values (−13% to −18%), consistent with regional myocardial dysfunction. (**B**) Segmental longitudinal strain curves obtained from a left apical view. Marked heterogeneity in peak systolic strain is observed among myocardial segments, with reduced peak systolic deformation consistent with dyskinetic motion represented by the yellow line. (**C**) Color-coded strain map illustrating the heterogeneous myocardial deformation with regional impairment and mild mechanical dispersion. CVA** represents aortic valve closure.

LV global systolic function was subjectively preserved. However, regional inferior septal dyskinesia was identified and confirmed using two-dimensional speckle-tracking echocardiography (Figure 4). Strain analysis revealed a mildly reduced global longitudinal strain (GLS) value of −17%, indicating subtle impairment of longitudinal myocardial deformation despite preserved radial systolic performance and normal fractional shortening (FS). Segmental strain assessment demonstrated reduced deformation predominantly within the basal aspect of the proximal interventricular septum consistent with focal regional systolic dysfunction.

Doppler interrogation revealed moderate mitral regurgitation (MR) and mild tricuspid regurgitation (TR), with a derived systolic pressure gradient of 35 mmHg and estimated RA pressure of 20 mmHg, indicating a high probability of moderate pulmonary hypertension (PH). During episodes of IAVD, pulsed-wave Doppler interrogation of transmitral flow demonstrated absence of consistent atrial contraction waves and beat-to-beat variability in diastolic filling patterns. This finding reflected loss of effective AV synchrony and impaired atrial contribution to ventricular filling, likely exacerbating diastolic dysfunction.

Based on the structural and functional abnormalities, a presumptive echocardiographic diagnosis of NSCM phenotype was established. The presence of marked atrial enlargement, suspected diastolic dysfunction, regional systolic dysfunction and bicavitary effusions supported the presence of CHF. During simultaneous electrocardiographic recording, intermittent ventriculoatrial conduction was observed at higher HRs (~280 beats/min), increasing the suspicion of FJT.

Additional diagnostic tests, including abdominal ultrasonography (AUS), a complete blood count (CBC) and serum biochemistry, were performed with no relevant abnormalities detected. Fine-needle aspirations taken from both the liver and spleen were unremarkable. Serology testing using an enzyme-linked immunosorbent assay (ELISA) was negative for FIV and FeLV. Serum cTnI measured by a highly sensitive quantitative assay at an external laboratory was increased (154.6 pg/mL, Ref. 5.0–60.0), consistent with significant myocardial injury. Although acromegalic cardiomyopathy was considered unlikely given the blood tests and physical examination, serum somatomedin C was measured due to the large size of the cat, which was found to be within normal limits (175 ng/mL, Ref. 40–180). Thyroid testing was performed due to the patient’s age and evidence of cardiac disease and was ruled out by a normal serum total T4 concentration (2.1 µg/dL, Ref. < 2.8).

Based on the clinical, electrocardiographic and echocardiographic findings, a presumptive diagnosis of biventricular CHF associated with a non-specific cardiomyopathy (NSCM) phenotype, pulmonary hypertension, and concurrent FJT with type I synchronization and IAVD was made. The cat was also considered at high risk of ATE. 

**Figure 5:**
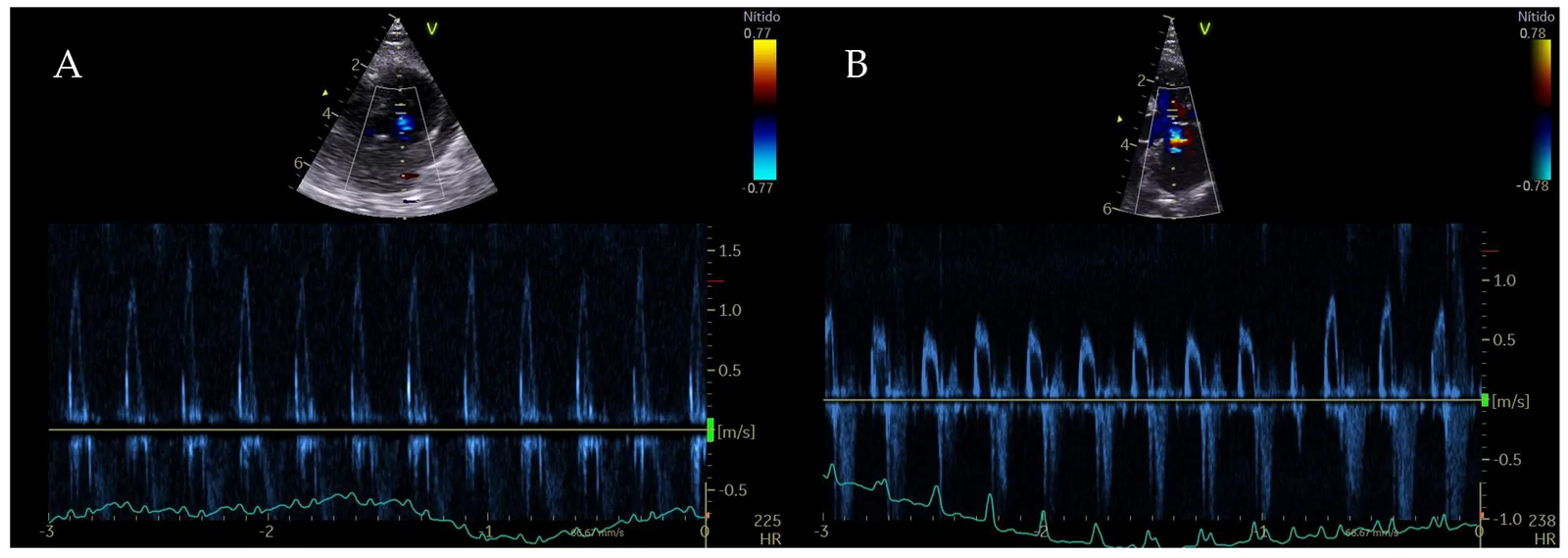
Pulsed-wave spectral Doppler evaluation of the left ventricular diastolic function on first echocardiographic evaluation (**A**) and just prior to discharge (**B**), when the patient was clinically stable. To note is the reduction in left ventricular filling velocities from 1.5 m/s to 0.7 m/s. Synchronous ECG showed type II (**A**) and type I IAVD (**B**).

The cat was hospitalized with oxygen supplementation and administered enoxaparin (1 mg/kg q12h SQ), furosemide (1 mg/kg q8h IV) and sildenafil (1 mg/kg q12h PO). Continuous RRR, HR, rhythm, NIBP, diuresis and water intake was performed for 24 h. The temperature remained between 38.1 °C and 39.5 °C; otherwise, the cat was bright and active. An intermittent gallop sound without a murmur was detected, with a HR of 224 beats/min. The ECG demonstrated an incessant FJT with normal NIBP. Resolution of the PH, pleural, and pericardial effusions was observed with significant decrease in both LA:Ao ratio and LV filling pressures (Figure 5). After 36 h, the cat was eating and stable to be discharged with oral therapy consisting of clopidogrel (18 mg q24h), furosemide (1.25 mg/kg q12h), sildenafil (1 mg/kg q12h), and benazepril (0.31 mg/kg q24h) combined with spironolactone (2.5 mg/kg/d).

Follow-up was scheduled two weeks after discharge once flow-cytometry results were received. A normal RRR (<30 breaths per minute) and appetite were reported upon phone consultation a week after discharge. However, the cat was readmitted 4 days later due to recurrence of tachypnoea at rest. ECG evaluation on readmission revealed a HR of 260 bpm with frequent isolated ventricular premature complexes (VPCs) exhibiting right bundle branch block (RBBB) morphology. A right caudal lung lobe infiltrate consistent with focal alveolar patterns was observed on thoracic radiographs, suggestive of cardiogenic pulmonary edema. Echocardiography revealed reduction in myocardial thickness and recurrence of mild pleural and pericardial effusions without cardiac tamponade. Comparative echocardiographic variables are shown in Table 1. Thoracocentesis was performed and 100 mL of serous pleural effusion was removed (2.8 g/dL TS). Biochemistry revealed a serum creatinine of 1.8 mg/dL (Ref. 0.8–2.4), but was otherwise unremarkable. Due to the concern of potential diuretic resistance, furosemide was up-titrated to 1 mg/kg q8h SQ until complete clinical stabilization in 24 h.

**Figure 6:**
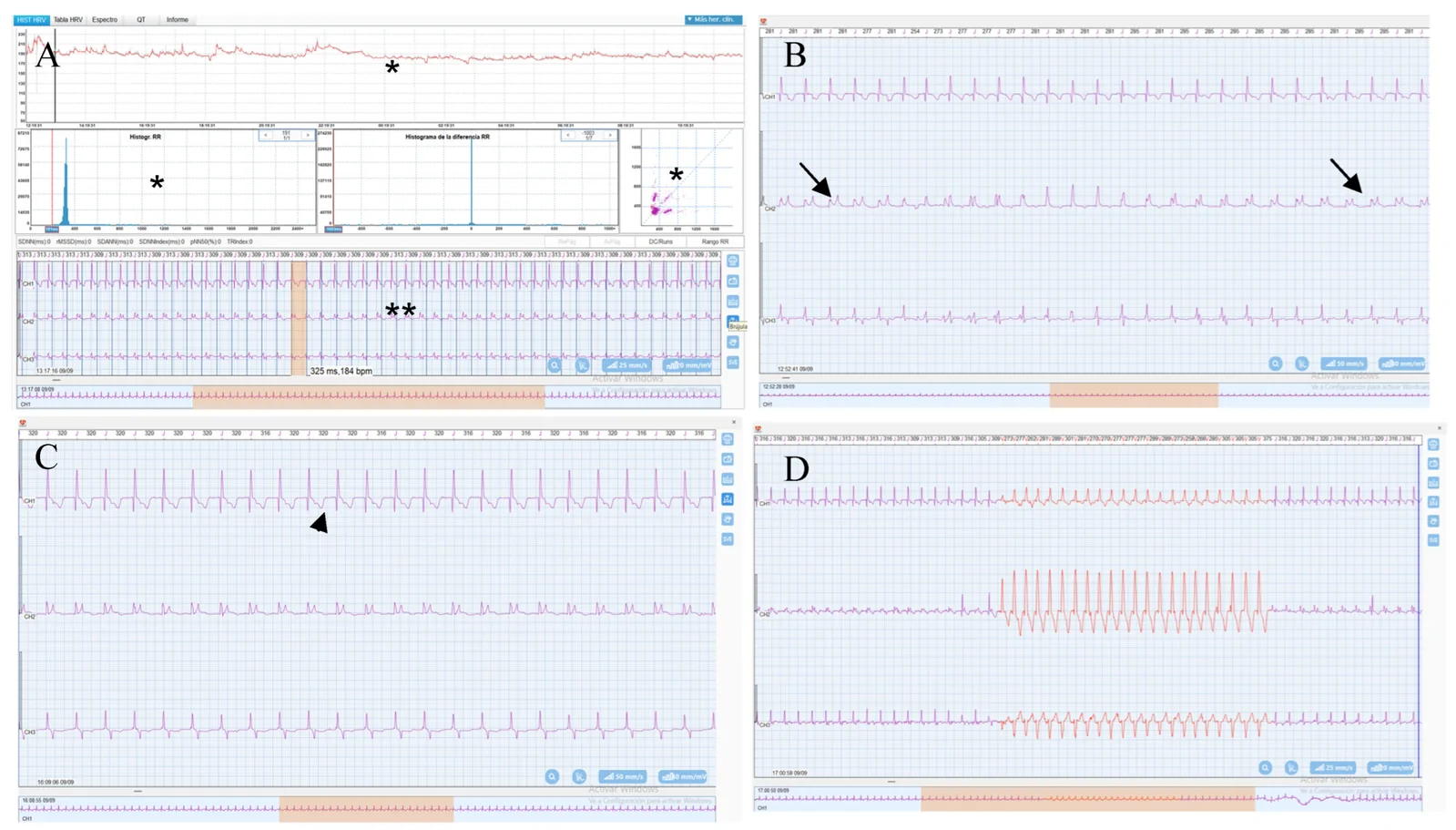
Holter monitoring in the hospital setting. (**A**) The HR during the recording is represented by the tachogram in red color (*). The y-axis represents the coupling interval and the x-axis timing. The predominant rhythm was an incessant narrow QRS complex arrhythmia with warm-up and cool-down phenomena and cycle length irregularity (**). The global HR variability was considered reduced, as shown in the histograms, based on the difference between R-R intervals, and the poincaré diagram (*). (**B**) When sinus impulses predominated during periods of fixed R-AD intervals (long arrows), a type II synchronization was suspected. (**C**) At higher HR of 240 beats/min, retrograde ventriculoatrial conduction with 1:1 ratio was suspected, raising the suspicion of FJT (short arrow). (**D**) A total of 114 VPCs were detected, mainly isolated (59/114), with two in monomorphic couplets (coupling interval 225 ms) and one triplet (coupling interval 250 ms). Occasional fusion and capture beats were also suspected. The most complex ventricular arrhythmia was recorded during manipulation exhibiting wide-QRS complex tachycardia at 257 beats/min with coupling interval of 233 ms, and another longer run for 6 s at 232 beats/min (coupling interval 258 ms) compatible with an accelerated idioventricular rhythm (AIVR) or ventricular tachycardia less likely based on HR, highlighted in the recording by red color.

On the following day, 24 h Holter monitoring was performed in hospital to better characterize the arrhythmia and to assess the frequency and complexity of ectopic beats (Figure 6).

The mean (215 beats/min), maximum (240 beats/min) and minimum (196 beats/min) HRs overlapped with values reported for cats in the hospital setting, accompanied by markedly reduced HR variability. Retrograde P’ waves inscribed in the terminal portion of the QRS complexes (pseudo-S waves) were observed, strongly suggestive of active 1:1 ventriculoatrial retrograde conduction (Figure 6).

Based on the clinical presentation and Holter monitoring following ECG assessment, an FJT was considered the most likely diagnosis. The main differentials were an accelerated AV junctional rhythm as this rhythm also displays gradual onset and termination but typically at lower ventricular rates (from 60 to 130 beats/min). An atrio-Hisian accessory pathway-mediated tachycardia based on the short PQ interval was also included, but it was considered less likely since it usually shows wider QRS complexes [50]. Other possibilities, such as orthodromic atrioventricular reciprocating tachycardia (OAVRT) or pre-excited focal atrial tachycardia (PEFAT) were considered unlikely based on the absent delta wave or ventricular pre-excitation during sinus rhythm and the presence of IAVD.

**Figure 7:**
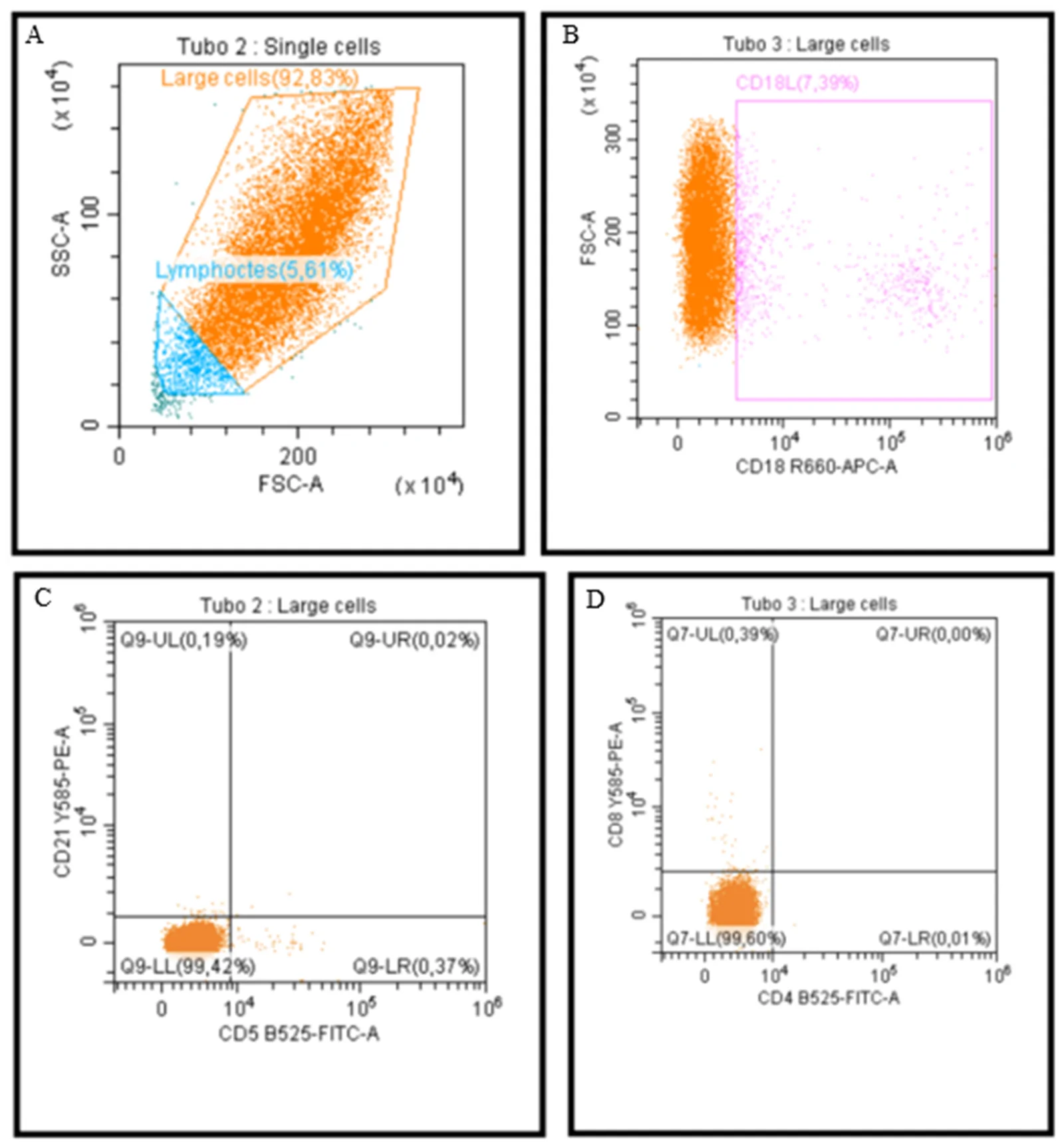
Flow cytometric immunophenotyping of pleural fluid cells obtained at the first presentation. (**A**) Forward and side scatter (FSC/SSC) gating on single nucleated cells identified a predominant population of large cells with high internal complexity (92.8%) and a minor population of small lymphocytes (5.6%). (**B**) Within the large-cell gate, a minimal percentage (7.4%) of cells expressed CD18. (**C**) Large cells lacked expression of CD5 and CD21, with most events located in the double-negative quadrant. (**D**) Similarly, no significant expression of CD4 or CD8 was detected, revealing a phenotypically negative (‘null-cell’) profile that initially raised clinical suspicion for an atypical lymphoid malignancy. No phenotypically distinct T- or B-cell population was identified.

Following gating on single nucleated cells to exclude debris and doublets by the external laboratory, flow-cytometry results of the pleural fluid returned negative for all biomarkers (CD18, CD21, CD5, CD4, CD8), suggesting atypical (neither B nor T) lymphoproliferative disorder (e.g., atypical phenotype lymphoma) (Figure 7), but further testing for confirmation (e.g., polymerase chain reaction (PCR)-based clonality assessment of antigen receptor gene rearrangements, PARR) was declined by the owner.

Since the patient was deemed clinically stable, a thoracoabdominal CT scan was performed to stage a potential mediastinal lymphoma. The CT findings were unremarkable except for cardiomegaly with mild pericardial effusion and pleural effusion at the level of the ventral aspect of both cranial lung lobes.

**Figure 8:**
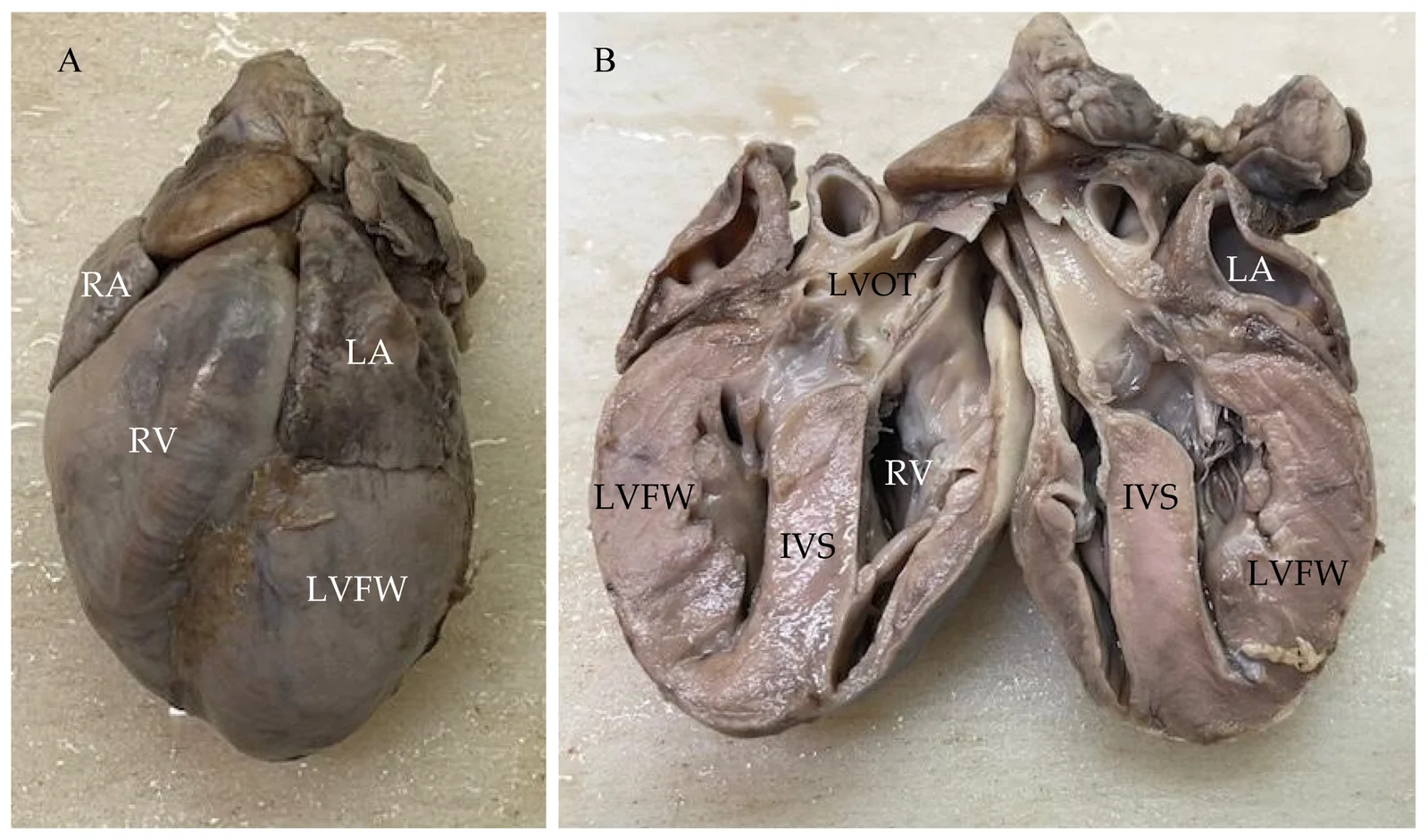
Gross pathology of the heart after formalin fixation. (**A**) External view demonstrating marked global cardiac enlargement, globose morphology, and severe biatrial dilatation. (**B**) Longitudinal section (four-chamber view) showing severe left ventricular hypertrophy, interventricular septal thickening, pale myocardial discoloration, and left atrial dilation in a heart fixed in a systolic contraction state. Morphometric measurements: base-to-apex length, 6.7 cm; heart base width, 4.0 cm; RV free wall thickness, 5 mm; LVFW thickness, 14 mm; IVS thickness, 11 mm; RA wall thickness, 2 mm; and left atrial (LA) wall thickness, 3 mm. LA, left atrium; LVFW, left ventricular free wall; LVOT, left ventricular outflow tract; IVS, interventricular septum; RA, right atrium; RV, right ventricle.

The cat was hospitalized and furosemide was down-titrated (1 mg/kg q12h SQ), while the remaining CHF therapy was left unchanged. Unfortunately, 36 h later, the cat experienced a vomiting episode followed by cardiorespiratory arrest and spontaneous death. A postmortem examination was performed (Figure 8).

Gross evaluation of the formalin-fixed heart revealed global cardiac enlargement with marked biatrial dilatation, diffuse myocardial paleness, and severe concentric biventricular hypertrophy in a systolic contraction state (Figure 8). Sectioning along the long axis demonstrated marked luminal narrowing with diffuse thickening of the ventricular walls and interventricular septum, combined with biatrial dilation (Figure 8B). Gastrointestinal content within the mainstem bronchus and pneumonia was present, which, based on these findings, was likely secondary to aspiration.

**Figure 9:**
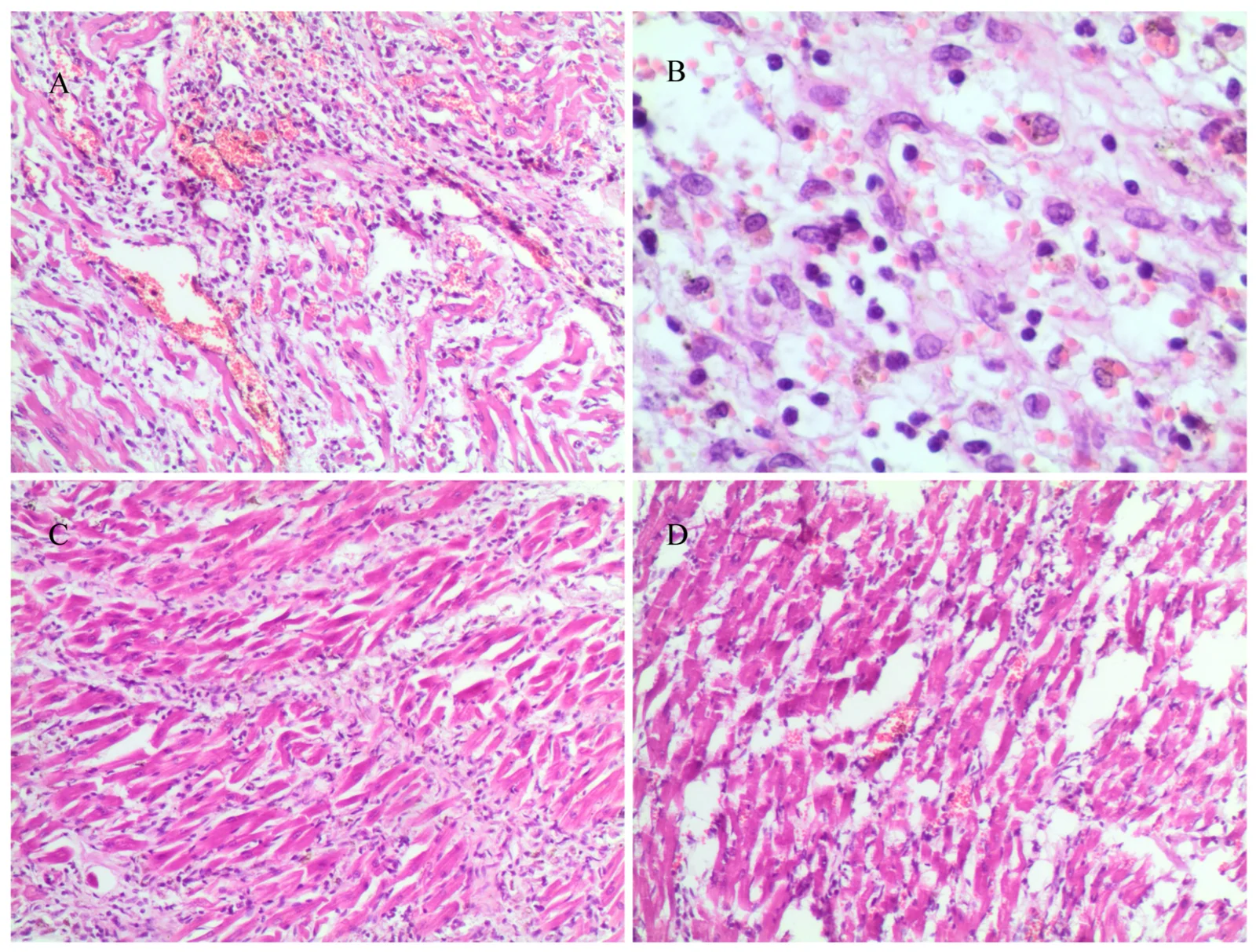
Representative photomicrographs of the histopathological findings. (**A**) Left atrial myocardium (100× magnification) demonstrating diffuse interstitial edema and inflammatory cell infiltration widely separating cardiac muscle fibers. (**B**) High-power view of the atrial wall (400× magnification) showing a mixed, predominantly mononuclear inflammatory infiltrate composed of mature lymphocytes, macrophages, and occasional plasma cells dissecting the interstitium alongside marked vascular congestion. (**C**) Right ventricular myocardium (100× magnification) and (**D**) left ventricular myocardium (100× magnification) exhibiting similar interstitial inflammatory cell infiltration, congestive changes, and myocardial bundle dissociation. Hematoxylin and eosin (H&E) stain.

Histopathological examination (Figure 9) confirmed diffuse, mononuclear myocarditis affecting both the ventricles and atria, as well as the pericardium and subepicardial adipose tissue. The inflammatory infiltrate was predominantly composed of mature lymphocytes, macrophages, and occasional plasma cells, accompanied by vascular congestion and mild interstitial fibrosis. No infectious microorganisms or neoplastic cells were identified in any of the examined tissue sections.

## 3. Discussion

To the best of the authors’ knowledge, this is the first reported case of suspected FJT associated with diffuse lymphocytic myocarditis of undetermined etiology in a cat presenting with clinical signs of CHF.

The ECG diagnosis of FJT in humans includes a HR > 100 beats/min [8]. In dogs, a median HR of 140 beats/min has been stated and considered tachycardia since the pacemaker cells in the AV node range from 40 to 60 beats/min [2]. In cats, there remains a lack of established reference criteria for this rhythm; however, our patient maintained a HR above 200 beats/min.

Healthy cats monitored in a hospital setting display marked sympathetic activation, yielding mean heart rates ranging up to 187–206 beats/min and reduced HR variability [51]. Although the mean HR in our patient was slightly higher than these reported values in healthy hospitalized cats [51], absolute HR in this upper range can still overlap between individuals with stress-induced sinus tachycardia and the suspected FJT in this patient. Because HR alone was insufficient to characterize the arrhythmia in our case, the diagnosis of FJT relies on broader criteria.

Based on the existing literature across species [1,2,8], we propose the following diagnostic criteria for identifying suspected FJT in cats: (1) persistent narrow QRS complex tachycardia of supraventricular origin; (2) discrete cycle length irregularity with gradual ‘warm-up’ and ‘cool-down’ behaviors on 24 h Holter monitoring; (3) absence of initiation by a critical coupling interval or premature ectopic beats, ruling out classical re-entrant mechanisms; and (4) concurrent presence of type I IAVD, reflecting independent sinus and junctional pacemaker rates.

Conversely to some types of SVTs reported in cats, the FJT was not precipitated by extrasystolic beats, suggesting a lack of dependence on a critical coupling interval for tachycardia initiation, similar to what it is described in human patients [52]. Furthermore, the variability in cycle length and the presence of acceleration–deceleration phenomena made re-entry an unlikely underlying mechanism, including re-entrant SVTs, such as AV nodal reciprocating tachycardia (AVNRT) and OAVRT [4,15,52,53]. Although adenosine has been used to help differentiate AV nodal-dependent from AV nodal-independent narrow QRS complex SVTs, it was not administered in this case because studies in dogs have demonstrated inconsistent effects on AV nodal [53], and comparable studies in cats are lacking.

Accelerated AV junctional rhythm (so-called non-paroxysmal junctional tachycardia) was considered a differential diagnosis due to its gradual onset and termination. However, it usually exhibits P’ waves preceding the narrow QRS complexes with P’Q intervals shorter than the sinus PQ interval [1]. Furthermore, the accelerated AV junctional rhythm is attributed to enhanced normal automaticity, whereas FJT may also arise from triggered activity [1,2].

Sympathetic nervous system activation in the setting of CHF could have reduced the activation threshold of action potentials as a result of enhanced automatism on the sinus node and the AV junction. This was also favored by the decreased HR variability determined by Holter assessment, as reported in human patients with FJT [54].

Triggered activity due to delayed afterdepolarizations (DAD) as a result of ischemia, edema and inflammation affecting the bundle area was considered a plausible electrogenic mechanism for the arrhythmia based on histopathological findings. In addition, the presence of underlying myocardial inflammation was considered an excellent anatomical substrate for arrhythmia perpetuation by causing intracellular Ca^2+^ overload, similar to humans with FJT [54]. Here, increased Ca^2+^ release from the sarcoplasmic reticulum favors a transient inward current of Na^+^ resulting in premature depolarization of the cell [55].

In humans, FJT can be initiated by rapid atrial or ventricular pacing or by isoprenaline infusion, but not by programmed electrical stimulation unless triggered activity is present [3,56]. Therefore, only detailed electrophysiological mapping can determine the exact underlying electrogenic mechanism [2,53]. In cats, these procedures are not readily available, and their feasibility is further limited by small patient size and concurrent decompensated CHF, precluding definitive confirmation of the arrhythmia diagnosis in this case.

In dogs, FJT with IAVD can lead to significant changes in cardiac hemodynamics due to the lack of normal sequential AV electrical and mechanical activity [2]. Since AV synchrony and coupling are impaired, a drop in stroke volume may be expected, particularly if concurrent severe LV systolic dysfunction develops [2].

During FJT, atrial depolarization may arise from the sinus node pacemaker or via retrograde ventricular–atrial conduction [1]. Retrograde ventricular–atrial activation occurs when the junctional pacemaker is faster than the sinus node pacemaker, resulting in retrograde activation of the atrial myocardium and overdrive suppression [1]. This was observed during periods of stress during manipulation or active CHF in our patient. 

It has been demonstrated that, when the P waves appear after the QRS complexes, the systemic blood pressure falls, and the RA pressure rises [57]. Doppler interrogation of pulmonary venous flow was not performed in this case. In human medicine, pulmonary venous Doppler has been shown to be a useful adjunct in the assessment of junctional arrhythmias [58]. During junctional rhythms, simultaneous atrial and ventricular systole leads to large early systolic flow reversals in the pulmonary veins noted with every beat [58]. In our case, the most important hemodynamic changes occurred when the atria contracted against closed AV valves as seen during the echocardiogram. This explains the larger right and left atrium observed on echocardiography, prior to initiation of therapy for the CHF, where increased automaticity due to the high sympathetic tone was present. 

The mild hypotension on admission could at least be partially explained by the loss of the atrial contribution to ventricular filling, accelerating the firing rate of the sinus node in an attempt to re-establish a more physiologic AV sequence. In addition, the sinus discharge rate varies inversely to the arterial pressure via the baroreceptor reflex [57]. Another possibility is that IAVD type I leads to simultaneous atrial–ventricular contraction, so the content of the RA cannot be emptied into the RV, explaining the volume overload. When ventricular–atrial conduction was visualized, the discharge rate of the sinus node decreased so the atrial myocardium could be captured by the retrograde depolarization wavefront originating from the AV junction and therefore activated in a concentric retrograde fashion. The position of the retrograde P’ wave in the descending portion of the R wave or ST segment has been demonstrated to be highly dependent upon the retrograde conduction velocity along the AV junction [57].

Lymphocyte-rich pleural effusions have been reported in cats with RCM, HCM, ARVC and several neoplastic processes including mediastinal lymphoma and thymoma [59]. These effusions have a milky appearance and must be differentiated from chylous effusions, which are characterized by higher triglyceride concentrations in the effusion than in the serum and an effusion triglyceride:cholesterol ratio > 1 [59]. Chylothorax has been recognized in cats with several cardiac diseases including heartworm, although it can also be congenital, neoplastic (mediastinal lymphoma), traumatic or idiopathic [60]. Triglycerides and cholesterol were not measured in the pleural fluid of this patient as the fluid analysis was consistent with an exudate. Based on the presence of lymphoblasts in the fluid cytology and the patient’s poor response to standard CHF therapy, an atypical cardiac lymphoproliferative disorder was considered as a primary differential diagnosis antemortem.

Lymphoma is the most common hematopoietic tumor in cats [61]. This type of cancer constitutes 41% of all malignant tumors and 90% of hematopoietic tumors observed in cats [62]. It has been reported either as a primary (extranodal form) [63] or, more often, as an extension for multicentric or mediastinal forms [64]. The most common is gastrointestinal lymphoma, followed by extranodal, mediastinal, and multicentric lymphoma [65]. Primary cardiac lymphoma is infrequently reported in cats, being more likely metastatic carcinomas from pulmonary, salivary, oral or mammary glands [61,63]. Positive serology for FeLV or FIV has frequently been reported in cats with primary cardiac lymphoma [29,66]; however, neither was detected in the present case. In cases with multicentric lymphoma with cardiac involvement, cardiovascular signs are uncommon in cats and, if CHF occurs, pericardial effusion is often well tolerated even if moderate [64].

Diagnosis of lymphoma may involve techniques such as fine-needle aspiration (FNA) cytology, histopathological examination, immunophenotyping, flow cytometry, and molecular diagnostic techniques [65]. In the literature, diagnosis of cardiac lymphoma in cats is commonly reached antemortem by thoracic fluid or myocardial cytology [67,68,69]. Although few studies have demonstrated the utility of flow cytometry in supporting the diagnosis of feline lymphoma [31,70], flow cytometry was not definitive in the present case. Furthermore, one study suggested that flow cytometry alone may not be sufficient to confirm a diagnosis of lymphoma, particularly in cats with mediastinal lymphoma [71,72].

Although most large-cell lymphomas can be diagnosed by cytology, histopathological examination and/or more advanced molecular testing may be required [71,72,73]. However, diagnostic interpretation can be challenging due to limitations in the quantity and quality of lymphocytic infiltrates [65]. The suspected cardiac involvement limited the feasibility of obtaining an antemortem biopsy in the present case. Furthermore, distinguishing between reactive and malignant changes can be difficult on cytology or histopathology alone, and additional testing is often necessary. PARR can be a complementary tool to further confirm lymphoma in cats based on clonality [65]. However, it is crucial to supplement this information with morphology, immunohistochemistry (IHC), flow cytometry, and clinical indicators [74]. Molecular clonality analysis is used to diagnose atypical, mixed, or mature lymphoid proliferations, even with minimal sample quantities or in cases where the tissue structure is absent [65]. Given the unconclusive flow-cytometry results, performing a PARR clonality assay may have provided additional diagnostic information and strengthened the diagnostic interpretation in our case.

However, this technique also has some limitations especially in cats, including false-positive and false-negative results, and the need for careful handling and processing of the samples [65]. Both the quantity and quality of the samples are crucial for obtaining reliable results, particularly when pleural fluid is submitted [65]. In one recent study in cats, PARR performance in pleural fluid often exhibited numerous non-amplifications attributable to paraffin defects and DNA damage [65]. Furthermore, the presence of a chronic inflammatory environment, in which potentially neoplastic lymphocytes may have been mixed with reactive lymphocytes, could have further complicated the diagnosis of lymphoma in this case. These limitations have important implications for clinical decision-making, as treatment strategies differ substantially among cardiac lymphoma, primary cardiomyopathies, and myocarditis of varying etiologies. In our case, although PARR was offered, this test was declined by the owner due to financial constraints and the patient’s clinical deterioration.

One diagnostic challenge leading to the initial presumed diagnosis was flow cytometric immunophenotyping of the pleural effusion, which revealed a predominant population of large cells lacking T- and B-cell markers (CD5, CD21, CD4, and CD8), displaying a ‘null-cell’ profile. While such atypical immunophenotypes in cavitary effusions commonly prompt suspicion of a non-T/non-B lymphoproliferative disorder, no neoplastic cells were identified in the examined tissues postmortem. Based on our findings, severe lymphocytic myocarditis can generate reactive lymphocytic effusions that closely mimic cardiac lymphoma antemortem.

Myocarditis has been published in cats with HCM phenotype [31]. From a structural standpoint, the marked myocardial thickening and heterogeneous appearance observed on echocardiography in our cat—classified as an NSCM phenotype—correlated postmortem with diffuse lymphocytic myocarditis with pericardial involvement. These structural findings highlight the potential for inflammatory cardiomyopathy to mimic primary cardiomyopathies, as diffuse inflammatory cell infiltration and associated myocardial edema can result in myocardial pallor and apparent ventricular wall thickening [31].

Echocardiography on presentation showed LV-concentric hypertrophy with a more heterogeneous appearance in the follow-up scans performed later. It is possible that subclinical myocardial ischemia secondary to the tachyarrhythmia contributed to the heterogeneous myocardial appearance observed on follow-up echocardiography. In addition, long-standing tachyarrhythmias may lead to changes in wall thickness alongside systolic dysfunction (so-called ‘tachycardia-induced cardiomyopathy’) [75]. These cases typically exhibit features consistent with a dilated cardiomyopathy phenotype [75], which were absent in the present case. However, conventional endocardial indices of LV systolic function, such as FS, are well known to have limitations in feline cardiology, which can mask intrinsic myocardial impairment [76].

In contrast, speckle-tracking echocardiography can assess direct myocardial deformation independent of geometric assumptions [76]. Strain echocardiography performed in this cat showed hypokinesia affecting the proximal portion of the interventricular septum, consistent with regional myocardial systolic dysfunction. This regional mechanical pattern could also be compatible with ventricular interdependence secondary to RV pressure overload, resulting in altered septal contribution to LV systolic mechanics [76]. The discrepancy between normal-to-hyperdynamic FS values and mildly reduced GLS likely reflected early or subclinical LV systolic dysfunction undetectable by conventional methods or subjective evaluations. Previous studies in cats with HCM have shown abnormal systolic deformation of the myocardium affecting both the IVS and LV free wall [76], but studies in cats with myocarditis are still lacking. These findings suggest that incorporating advanced echocardiographic techniques into the diagnostic evaluation of cats with suspected myocarditis may help facilitate earlier detection of subclinical regional or global systolic dysfunction.

Although there was no clear evidence of systemic infection with myocardial involvement by an infectious agent, an infectious etiology cannot be definitively excluded in this cat. Testing was limited to retroviral screening; only feline FIV and not FeLV has been associated with myocarditis in cats [30]. Myocarditis has also been reported in association with FIP [32,77], but in this case no other clinical findings compatible with this syndrome were documented. Moreover, the cytological appearance of the effusion was compatible with lymphoma, and therefore FCoV PCR was not pursued either antemortem or postmortem. Histologically, FIP-associated myocarditis typically shows pyogranulomatous infiltration [32], which was not observed in this case. No clinicopathological criteria suggestive of feline panleukopenia virus (FPV) infection were identified, such as gastrointestinal signs or severe neutropenia [77,78]. Parvoviral myocarditis is well recognized in puppies [79], and early reports speculated about inclusion-body myocarditis in kittens [80]. Furthermore, parvoviral DNA was detected in myocardial tissue from cats with cardiomyopathy, raising the possibility of a role for parvovirus in feline cardiac disease [31]. However, recent molecular investigations have refuted a causal association between FPV and feline myocarditis [78].

Lymphocytic, histiocytic, and plasmacytic myocardial infiltrates have been described in cats with *Toxoplasma gondii* infection [81,82]. Moreover, transient myocardial thickening associated with T. gondii-positive status and myocardial injury has been reported as premortem [37,38]. However, in the present case, *T. gondii* infection was considered unlikely given the cat’s strictly indoor lifestyle and absence of hunting behavior, which characterizes most documented cases; therefore, testing was not prioritized. *Bartonella henselae* infection has been associated with myocarditis in cats, typically featuring myocardial infiltration by lymphocytes, histiocytes, and rare neutrophils [83]. In this case, postmortem PCR testing of the myocardium for *Bartonella henselae* was recommended but declined, and cannot be completely excluded.

Circulating cTnI, a biomarker of myocardial damage, has also been reported to be consistently high in a variety of myocardial diseases, particularly in myocarditis and myocardial infarction [35,84]. In the present case, the elevation in cTnI may have reflected myocardial injury associated with the inflammatory cell infiltration confirmed postmortem, or myocardial hypoxia or ischemia secondary to the tachyarrhythmia and ongoing CHF.

In summary, the clinical presentation and disease progression, together with the antemortem diagnostic findings and postmortem examination, illustrate the complexity of this case, characterized by recurrent episodes of CHF that remained refractory to medical management. This poor response to treatment was likely attributable to the challenges of adequately suppressing the arrhythmia while simultaneously managing the underlying myocardial inflammation. As seen in the recent human literature [29,85,86,87], the concurrent myocardial and pericardial involvement in this cat provides a plausible explanation for the refractory clinical course. These findings should be interpreted within the context of the inherent diagnostic limitations, including the lack of invasive electrophysiological mapping, antemortem endomyocardial biopsy, dedicated serial histopathology of the specialized conduction system, and broad-panel molecular screening for other infectious pathogens.

## 4. Conclusions

This case report represents the first description of a suspected FJT in a cat with type I IAVD associated with a diffuse, lymphocytic myocarditis of undetermined etiology. In addition to myocardial lymphoma, myocarditis must be included as a differential diagnosis in cats with lymphocytic effusions and cardiac abnormalities on echocardiography mimicking primary cardiomyopathies such as NSCM. The presence of apparently preserved global systolic function should be further interrogated by advanced speckle-tracking strain echocardiography to help detect subclinical regional myocardial impairment in cats with suspected inflammatory myocardial disease, alongside biomarker evaluations such as cTnI. Further research is needed to identify effective therapeutic strategies for managing junctional tachycardias in patients with concurrent CHF, with the aim of achieving adequate rate control and preventing progressive hemodynamic deterioration.

## Figures and Tables

**Table 1:** Selected echocardiographic variables obtained from two-dimensional (2D) echocardiography at presentation and follow-up.

Echo Variable	Admission (Day 1)	Re-Evaluation (Day 11)	Final Assessment (Day 15)
IVSd (mm)	8	8	6
LVFWd (mm)	8	8	7
LA:Ao ratio	3.17	1.81	1.84
FS (%)	55	68	71
Fused E/A waves (m/s)	1.5	0.7	1.5

Fused E/A waves reflected a single, merged transmitral inflow velocity wave resulting from tachycardia combined with dynamic isorhythmic AV dissociation (alternating type I and type II IAVD patterns). The lack of sustained electromechanical AV synchrony prevented separate resolution of early rapid filling (E wave) and active atrial contraction (A wave), representing a major limitation for conventional Doppler echocardiographic assessment of left ventricular diastolic function. IVSd, interventricular septum in diastole; LVFWd, left ventricular free wall in diastole; LA:Ao ratio, left-atrium-to-aortic-root ratio; FS, fractional shortening; IAVD: isorhythmic atrioventricular dissociation. FS was obtained from 2D loops in short-axis views.

## Data Availability

The original contributions presented in the study are included in the article; further inquiries can be directed to the corresponding author.

## Abbreviations

The following abbreviations are used in this manuscript:

AH	Atrial-Hisian interval
ARVC	Arrhythmogenic right ventricular cardiomyopathy
ATE	Arterial thromboembolism
AUS	Abdominal ultrasound
AV	Atrioventricular
AVNRT	Atrioventricular node re-entrant tachycardia
BCS	Body condition score
CBC	Complete blood count
CHF	Congestive heart failure
CT	Computed tomography
cTnI	Circulating troponin I
DAD	Delayed afterdepolarizations
ECG	Electrocardiogram
EF	Ejection fraction
FCoV	Feline coronavirus
FeLV	Feline leukemia virus
FIP	Feline infectious peritonitis
FIV	Feline immunodeficiency virus
FJT	Focal junctional tachycardia
FNA	Fine-needle aspiration
FPV	Feline parvovirus
GLS	Global longitudinal strain
HCM	Hypertrophic cardiomyopathy
HIV	Human immunodeficiency virus
HR	Heart rate
IAVD	Isorhythmic atrioventricular dissociation
IHC	Immunohistochemistry
IM	Intramuscular
IV	Intravenous
IVS	Interventricular septum
LA	Left atrium
LA:Ao	Left atrium to aortic ratio
LV	Left ventricle
MEA	Mean electrical axis
NIBP	Non-invasive blood pressure
NSCM	Non-specific cardiomyopathy
OAVRT	Orthodromic atrioventricular reciprocating tachycardia
PARR	Polymerase chain reaction for antigen receptor rearrangements
PCR	Polymerase chain reaction
PEFAT	Pre-excited focal atrial tachycardia
PH	Pulmonary hypertension
PO	Per os
POCUS	Point-of-care ultrasound
RA	Right atrium
RBBB	Right bundle branch block
RCM	Restrictive cardiomyopathy
RV	Right ventricle
SQ	Subcutaneous
SVT	Supraventricular tachycardia
TICM	Tachycardia-induced cardiomyopathy
TR	Tricuspid regurgitation
TS	Total solids
URL	Upper reference limit
VHS	Vertebral heart score
VPCs	Ventricular premature complexes
VPE	Ventricular pre-excitation
